# Mechanical ventilation using prime numbers: an exploratory study of numerical preferences and oxygenation

**DOI:** 10.1016/j.bjao.2026.100859

**Published:** 2026-09-11

**Authors:** Clemens R.M. Barends, Kai van Amsterdam, Douglas J. Eleveld, Jaap Jan Vos, Anthony R. Absalom

**Affiliations:** 1 Department of Anesthesiology, University Medical Center Groningen, Groningen, The Netherlands; 2 Department of Anesthesiology, Frisius MC, Leeuwarden, The Netherlands

**Keywords:** biases, heuristics, mechanical ventilation, medical decision making, prime numbers

Recent trends in studies on mechanical ventilation are towards the use of individualised, rational, and evidence-based ventilator settings to improve the outcome of the patient.^1^^,^^2^ Despite this trend to rationalise ventilator care, we have noticed a curious scenario that readers may recognise: when a colleague is asked to assist with the care for a mechanically ventilated patient, one of the first things the assisting colleague may do is adjust the ventilator parameters from odd to even numbers. Many of these changes are seemingly futile, no physiological explanation is given for the adjustments, and the changes are rarely undone. There is no evidence that such choices influence patient outcomes, and this phenomenon has not been explored quantitatively. The reason for this behaviour is unclear, but it suggests the existence of seemingly arbitrary, non-evidence-based choices made by medical professionals.

To examine whether the preference for even numbers has any inherent biomedical justification, we conducted a study using two uncommon but not unique complementary reasoning strategies: the argument from arbitrariness and *reductio ad absurdum*.^3^^,^^4^ The argument from arbitrariness questions whether the numerical preference is beneficial or perhaps merely habitual. In an extension of our argument from arbitrariness, we juxtapose the choice of even numbers with an equally arbitrary and seemingly absurd numerical set: the prime numbers. If an arbitrary set such as prime numbers is associated with better ventilatory outcomes, then the conventional preference for even numbers cannot be justified on biomedical grounds and a different explanation may be sought.

The current study investigated the hypothesis that there is a significant difference in the ratio of the peripheral oxygen saturation (Spo_2_) to the fraction of inspired oxygen (Fio_2_) (Spo_2_/Fio_2_ ratio) when prime numbers are used compared with even numbers for the parameters PEEP, plateau pressure (PPlat), and respiratory rate (RR).

## Methods

This was a retrospective cohort study. The local hospital review board waived the need for informed consent (30 June 2023 METc 2023/362, chair: Prof HPH Kremer). We extracted patient-agnostic data from the hospital electronic patient high-frequency data system from procedures under general anaesthesia during which positive pressure ventilation was applied. The system automatically records all anaesthesia data from ventilators at a minimum of 1 s intervals. We analysed PPlat, PEEP, and RR because they are recorded in volume- and pressure-controlled mode. Together, they offer a hypothetical comprehensive package for basic ventilator parameters. Only data points with all parameters (PPlat, PEEP, RR, Spo_2_, and Fio_2_) from individuals were used. Data from 10 operating rooms over 39 consecutive days (weekends and holidays excluded) were included. The dataset contained 900,273 data lines for the parameters Spo_2_/Fio_2_ ratio, PEEP, PPlat, and RR.

### Statistics

Data are summarised as medians (interquartile ranges [IQRs]) for descriptive purposes, whereas group comparisons were performed using a two-tailed Mann–Whitney *U*-test. The Spo_2_/Fio_2_ ratio was calculated from Spo_2_ and Fio_2_, both recorded as percentages. Statistical testing was performed in R (https://www.R-project.org/; 2018).

**Supplementary Fig 1:**
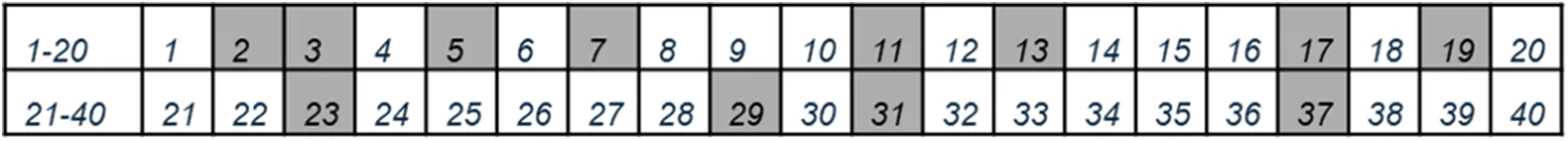
Eratosthenes sieve identifying prime numbers. Grey, prime number; white, non-prime number.

Most ventilator settings are in the range of 0–40 (cm H_2_O). Arguably, lower ventilatory pressure values are found more often in patients with fewer respiratory problems, reflected by a higher Spo_2_/Fio_2_ ratio. To compensate for a possible bias from the higher frequency of prime numbers in the lower range (0–20), we identified two number ranges with similar frequency distributions of prime and even numbers and equal mean values of prime and even numbers. Series 1 (4–14) was clinically relevant for PEEP, and series 2 (6–24) was clinically relevant for PPlat and RR (Supplementary Table 1). An Eratosthenes sieve identifying the prime numbers for both ranges is presented in Supplementary Fig. 1.

## Results

Data points for PEEP (prime, 566,504; even, 308,888), PPlat (prime, 260,496; even, 498,099), and RR (prime, 135,107; even, 657,493) were available for retrospective analysis. The Spo_2_/Fio_2_ ratio was significantly higher when PEEP (*P*<0.01), PPlat (*P*<0.01), and RR (*P*<0.01) were prime numbers than when they were even numbers. Median [IQR] scores and test results are presented in Table 1.

**Table 1:** Spo_2_/Fio_2_ ratio in prime- *vs* even-numbered ventilator parameters. Fio_2_, fraction of inspired oxygen; IQR, interquartile range; PPlat, plateau pressure; RR, respiratory rate; Spo_2_, peripheral oxygen saturation.

Ventilator parameter	Direction of effect	*P*-value	Prime, median (IQR)	Even, median (IQR)
PEEP	Prime>even	<0.01	2.53 (0.59)	2.32 (1.02)
PPlat	Prime>even	<0.01	2.47 (0.64)	2.48 (0.82)
RR	Prime>even	<0.01	2.51 (0.61)	2.50 (0.64)

## Discussion

In this study, we found that the use of prime numbers for ventilator parameters PPlat, PEEP, and RR is associated with a higher Spo_2_/Fio_2_ ratio than even numbers, indicating that less oxygen is needed to achieve a higher Spo_2_. A low ratio is used as a measure of respiratory compromise. These results may seem to suggest that the use of prime numbers for these ventilator parameters plays a role in improving oxygenation compared with the use of even numbers.

The choice to compare even numbers with prime numbers is based on the *reductio ad absurdum* and argument from arbitrariness methods of reasoning.^3^^,^^4^ The introduction of an alternative but equally arbitrary numerical set allowed us to highlight that the choice of even numbers offers no intrinsic advantage over other sets, and it explicitly demonstrates that the preference for even numbers lacks an inherent justification and instead may only reflect a cognitive or conventional habit. Juxtaposing another arbitrary integer set also raises fundamental questions about the underlying medical decision-making process. It stands to reason that even numbers will not benefit the patient more than uneven numbers or any integer set, yet in clinical practice this reasoning is seemingly often overlooked.

Although we could have chosen odd numbers, or any other integer set, we set out to reduce this comparison to the absurd. The prime numbers are thought to play important roles in a myriad of physical, chemical, and biological processes.^5^, ^6^, ^7^ This lends at least a modicum of reasoning (albeit slightly absurd) that prime numbers may also influence physiological processes, modulate patient outcome, and play a role in optimising ventilatory strategies.

The premise of this study was the observation that clinicians change the ventilator to even numbers. To an uninformed observer, the clinician setting the ventilator may seem to be decisive and to be making informed and intelligent choices. The reality is that these choices have no known physiological rationale, and the observation begs for an explanation. Many studies have shown how numbers are processed and what influence the form or value of the number has on our thoughts. Some numbers are easier to remember, and others influence the way we judge the items these numbers are associated with.^8^^,^^9^ When chance is involved, odd numbers and prime numbers seem to be preferred.^10^ When faced with a mentally demanding task, humans subconsciously lighten their mental loads by preferring cognitive processes that are less burdensome, and processing even numbers has been shown to require less effort compared with odd numbers.^11^, ^12^, ^13^ A plausible theory is that colleagues called upon for assistance and finding themselves faced with a situation already not controlled by the requester subconsciously start acting with simple tasks that provide a sense of agency and also lighten the cognitive burden of the situation at hand: they introduce more even numbers.

The results of this study should be interpreted with caution and with several caveats. Our study was retrospective and non-longitudinal. The patient-agnostic dataset contained multiple observations per patient, with unequal numbers of observations contributed by different patients, preventing patient-level aggregation, repeated-measures analyses, or mixed-effects modelling. Consequently, our results reflect patterns among observations rather than independent patient-level effects.

Second, this study was exploratory, and no sample size analysis was performed. With a dataset as large as ours, it is unsurprising to find a statistically significant result with a very small clinical effect size. Although the data suggest an association between the use of prime numbers and a higher Spo_2_/Fio_2_ ratio, we cannot draw conclusions about causality. Readers should take this into account when deciding whether to implement our findings in their clinical practice. The presence of hypothetical associations between prime numbers and biological and physical processes has served only as a prompt to choose this integer set for a comparison with even numbers. Other integer sets (Fibonacci sequences, Pell or Lucas numbers, Mersenne primes, etc.) may have different associations, although their roles in biological processes have received little attention.

In contrast to the conventional choice of even numbers, the use of prime numbers for PEEP, PPlat, and RR was associated with a statistically significantly higher Spo_2_/Fio_2_ ratio. The intentionally reductive nature of our approach illustrates how seemingly structured clinical decisions may arise from arbitrary or heuristic processes. Deriving normative conclusions for clinical practice from these findings and adopting prime numbers for ventilator settings would merely repeat the underlying arbitrariness. The observed differences should be regarded as a prompt for critical reflection rather than as a basis for clinical guidance.

One could argue that the choice of even numbers may free up mental capacity, which may facilitate execution of various other clinical tasks. Health care providers using mechanical ventilation should focus on biological and physiological reasoning when caring for their patients, and they should be aware of their subconscious preferences for parameter values. By choosing even numbers for the ventilator, we might unwittingly be caring for ourselves rather than for the patient. It is very important for caregivers to know that their decisions may be influenced by such heuristics and biases. This awareness should foster doubt about choices and conventions.

## Authors’ contributions

Conceptualisation: CB, JV, AA

Investigation: CB, KA, DE, JV, AA

Methodology: CB, KA, DE, JV, AA

Project administration: CB, JV

Resources: CB, JV

Supervision: CB, AA

Writing—original draft: CB, KA, DE, JV, AA

Writing—review and editing: CB, KA, DE, JV, AA

Approval of the submitted version: CB, KA, DE, JV, AA

Data curation: KA, DE

Formal analysis: KA, DE

Software: KA, DE

Visualisation: KA, DE

Validation: AA

## Funding

Department of Anesthesiology, University Medical Center Groningen, Groningen, The Netherlands.

## Declarations of interest

DJE is an editorial board member of the Anesthesiology journal. He has received travel expenses from BD. The other authors declare that they have no conflicts of interest.
