# Supplementary material for: Mechanical ventilation using prime numbers: an exploratory study of numerical preferences and oxygenation
Source: BJA Open. 2026 Sep 11;20:100859. doi: 10.1016/j.bjao.2026.100859 (PMC13598160; doi:10.1016/j.bjao.2026.100859)
Supplement: Supplementary file 1 [file mmc1.docx]

Supplementary Table 1:Number ranges for series 1 and 2

| **Series** | **Group** | **Numbers** | **Mean (sd)** | **Parameter** |
| --- | --- | --- | --- | --- |
| 1 | prime | 5,7,11,13 | 9 (3.65) | PEEP |
|  | even | 4,6,8,10,12,14 | 9 (3.74) | PEEP |
| 2 | prime | 7,11,13,17,19,23 | 15 (5.80) | PPlat and RR |
|  | even | 6,8,10,12,14,16,18,20,22,24 | 15 (6.05) | PPlat and RR |
| PEEP: Positive End Expiratory Pressure: PPlat: Plateau Pressure; RR: Respiration Rate | | | | |
